# The Use of Human Platelet Lysate (hPL) Enables 3D Cultivation of ADSCs Derived From Human Breast Tissue

**DOI:** 10.1155/sci/3165313

**Published:** 2026-09-22

**Authors:** Annelie Koenen, Annemarie Schroeder, Toralf Reimer, Guido Hildebrandt, Katrin Manda

**Affiliations:** ^1^ Department of Radiotherapy and Radiation Oncology, University Medical Center Rostock, Suedring 75, Rostock 18059, Germany, uni-rostock.de; ^2^ Johannes Wesling Klinikum Minden, Institut für Pathologie, Hans-Nolte-Straße 1, Minden 32429, Germany; ^3^ Comprehensive Cancer Center MV, Campus Rostock, Schillingallee 68, Rostock 18057, Germany; ^4^ Department of Obstetrics and Gynecology, University Medical Center Rostock, Suedring 81, Rostock 18059, Germany, uni-rostock.de

## Abstract

**Background:**

For the application of mesenchymal stem cells (MSCs) in regenerative medicine, xeno‐free ex vivo cultivation is necessary. Therefore, this study aimed to investigate whether adipose‐derived stem cells (ADSCs) from human breast tissue could be cultivated with human platelet lysate (hPL)—a promising alternative derived from human platelets—instead of fetal bovine serum (FBS).

**Methods:**

The influence of hPL within the culture medium of ADSCs from the female breast was analyzed regarding immunophenotype, adipogenic differentiation capacity, growth kinetics, and cell cycle distribution of ADSCs. Subsequently, the formation of spheres in hPL‐supplemented medium was characterized in detail.

**Results:**

In comparison to FBS, the supplementation of medium with hPL resulted in a change in morphology, but the immunophenotype remained unchanged. Furthermore, supplementation of the medium with hPL caused an improved adipogenic differentiation capacity, increased growth, and shortened population doubling time (PDT) in ADSCs. Additionally, the cultivation of ADSCs with hPL led to spontaneous formation of spheres.

**Conclusion:**

hPL is not only a replacement for the ethically controversial cell culture additive FBS but also offers a xeno‐free method for the cultivation of breast tissue‐derived ADSCs and thus represents an important step from bench to bedside.

## 1. Introduction

In 2002, adipose tissue was identified as a new source of mesenchymal stem cells (MSCs) [1]. The so‐called adipose‐derived stem cells (ADSCs) meet the criteria for the declaration of multipotent mesenchymal stromal cells, defined by the position statement of the International Society for Cellular Therapy (ISCT) in 2006 [2]. In detail, ADSCs are plastic adherent, express specific CD markers (CD105, CD90, and CD73), are capable of self‐renewal, and can differentiate into the osteogenic, chondrogenic, and adipogenic lineages [3]. So, they can be used for therapeutic purposes in regenerative medicine, like regeneration of the articular cartilage of the knee joint and the ear cartilage via implanted ADSCs in mice. The implanted ADSCs stimulate the tissue with cytokines and growth factors and enhance angiogenesis [4]. Similarly, bone marrow‐derived MSCs were able to differentiate into insulin‐producing cells in vitro. The injection of these differentiated cells into mice with induced diabetes resulted in reduced blood glucose levels and increased insulin levels [5]. These represent a promising application for cell‐based therapy. Particularly in the field of wound healing, for example, in diabetic mice, it can be further improved by the additional application of lipoic acid, leveraging its immunomodulatory and anti‐inflammatory effects [6]. This illustrates how ADSCs cultured in human platelet lysate (hPL) could support xeno‐free approaches in stem cell‐based therapies for diabetes or the regeneration of chronic wounds.

Usually, ADSCs are cultivated in a two‐dimensional (2D) culture. However, they can also be cultivated in 3D. In this case, cells grow as cell aggregates called spheres without contact with the culture dish. These techniques of cultivation enhance the differentiation capacity into adipocytes and osteocytes as well as the preservation of stemness of MSCs. Moreover, the anti‐inflammatory and immunomodulatory characteristics also increase, so the 3D cultivation can improve the use of MSCs in regenerative medicine [7, 8]. To generate the spheres, dynamic methods—such as the spinner flask method, with the help of centripetal force, the cells are brought to aggregation—and static methods are used, like the hanging drop method, the liquid overlay technique, or the use of low‐attachment plates, which allow the spontaneous formation of aggregates [9]. So far, the trigger for the spherical formation has not been found; a special subpopulation of MSCs called MUSE cells (multilinear‐differentiating stress‐enduring cells) is discussed as a possible initiator [10]. MUSE cells are double‐positive for CD105, a MSC marker, and stage‐specific embryonic antigen‐3 (SSEA‐3), a human pluripotent stem cell marker, so they can differentiate into all three germ layers with stress tolerance and without building malignant tumors [11, 12]. So, these cells can also be used for tissue regeneration [13].

In in vitro cell culture, serum is used as a source of growth factors, hormones, transport proteins, and other proteins required for cells to grow [14]. Currently, most researchers use fetal bovine serum (FBS) for the cultivation of cells, also of ADSCs [15]. The annual usage of FBS is approximately 500,000 L. FBS is obtained from calves of pregnant cows out of herds for meat production via cardiac puncture. At the time of puncture, the calves were at least 3 months old so that the heart was large enough [16].

Using FBS in in vitro culture offers several benefits, such as universal application. FBS supplies hormones, growth factors, lipids, and transport proteins cells needed for mitosis [14]. FBS is also a constant production because it is a byproduct of meat production [16]. However, there are many disadvantages in the use of FBS, like allergic reactions from patients under clinical applications [17]. FBS is a poorly known serum with batch‐to‐batch variations, and potential contamination with bacteria, viruses, fungi, yeast, and endotoxins is possible [18].

Likewise, the calves are not under anesthetic treatment during the puncture, which is an ethical problem [16]. Therefore, an alternative to FBS for in vitro cultivation is being sought. In 2005, Doucet et al. [19] described the use of hPL in the cultivation of MSCs. There are different methods known for the production of hPL from thrombocytes; the two most prevalent manufacturing methods are the platelet‐rich method or the buffy coat method [20].

There are some advantages in the use of hPL in cell culture, like a better replication of the natural microenvironment of the cells due to the human origin, and therefore, no allergic reactions by humans during a therapeutic treatment occurred. To produce hPL, expired blood products—such as whole blood and thrombocyte concentrates from blood banks—can be used, so there are no ethical problems in patient care with blood products. Another source of platelets can be leukoreduction filters, which are used while manufacturing whole blood transfusions [21].

Since the hPL culture medium is prone to gelatinization due to its fibrinogen content, which triggers blood clotting, heparin must be added to the medium as an antagonist [22]. A possible virus contamination of hPL can be excluded by two tests after the blood donation.

Investigations have shown that ADSCs derived from abdominal fat tissue can be successfully cultivated in a hPL culture medium [23–25]. To our knowledge, there are no studies to date on whether hPL is an alternative to FBS in the cultivation of ADSCs isolated from the human breast tissue. While ADSCs derived from breast tissue share a number of similarities with those derived from abdominal fat—such as comparable morphological and phenotypic characteristics [26], a similar cell‐surface phenotype [27], multilineage differentiation capabilities, and comparable gene expression profiles [28]—there are distinct differences in key characteristics between the two cell types. ADSCs from the breast tissue differ in some characteristics from ADSCs from abdominal adipose tissue. ADSCs isolated from different origins are influenced by their microenvironment and therefore possess distinct properties. For example, ADSCs from breast tissue exhibited higher colony formation potential and higher expression levels of osteocalcin in osteodifferentiated cells compared to those from the abdominal fat tissue [29]. Additionally, breast tissue‐derived ADSCs have superior immunomodulatory and antioxidant abilities compared to abdominal‐derived ADSCs [26]. Furthermore, ADSCs from breast adipose tissue demonstrated a greater self‐renewal potential in vitro than ADSCs from abdominal fat [29]. Therefore, it is assumed that they have a higher chance of maintaining and expanding their stem cell population as more cells remain in the undifferentiated state compared to ADSCs from abdominal fat tissue.

The aim of our study was therefore to investigate whether hPL also represents a suitable alternative to FBS for the in vitro cell culture of ADSCs from the human breast tissue. This would enable xeno‐free ex vivo culture, which is necessary for the application of breast‐derived ADSCs in regenerative medicine to avoid allergic reactions or contamination. To this end, we analyzed the influence of hPL compared to FBS as a supplement to the culture medium of ADSCs from the female breast on the immunophenotype, adipogenic differentiation capacity, growth kinetics, and cell cycle distribution of ADSCs. Furthermore, sphere formation in the hPL‐supplemented medium was characterized in detail.

## 2. Materials and Methods

### 2.1. Cell Cultivation

ADSCs were isolated from breast tissue of healthy women, which underwent a breast reduction, and were cryopreserved with 5% DMSO (Merck, Germany) at −150°C. The isolation of ADSCs was approved by the ethics committee at the University Medical Center Rostock, Germany (Registration Number: A2017‐0049); the isolation protocol has already been described in a former work [30]. For every experiment, pooled ADSCs from 10 donors were used. The culture medium contained DMEM/F12 (Thermo Fisher Scientific, USA) with 1% penicillin/streptomycin (Sigma Aldrich Chemie, Germany). Additionally, cells were supplemented with either 10% FBS (Biochrom GmbH, Germany) or 10% hPL and 2 U/mL heparin (both PL BioScience, Germany). Cells were incubated at 37°C and 5% CO_2_ with a medium change (mc) every third day with a seeding density of 5 × 10^5^ cells per T75 cm^2^ culture flask or 1.5 × 10^5^–2 × 10^5^ cells per T25 cm^2^ culture flask. According to manufacturer’s instructions, the hPL is won from multiple donors pooled in large batch sizes after a freeze–thaw process from platelet‐rich plasma (PRP) under aseptic conditions.

### 2.2. Immunophenotyping

Mesenchymal surface markers of ADSCs were analyzed using flow cytometry, as previously described [30]. Briefly, cells were trypsinized, washed with staining buffer (BD Pharmingen, Germany), and stained with antibodies against CD31‐PE, CD45‐PE, and CD106‐PE (Biolegend, UK) and CD29‐PE, CD34‐PE, and CD90‐FITC (BD Biosciences) on ice and in the dark. Nonspecific binding was determined using fluorochrome‐conjugated isotype control antibodies. After incubation with 7‐aminoactinomycin (7‐AAD, Biolegend), analysis was performed using a flow cytometer (Cytomics FC 500, Beckman Coulter); evaluation was performed using CXP software.

### 2.3. Adipogenic Differentiation Capacity

About 2.5 × 10^4^ ADSCs in passages 3, 4, and 5 were seeded per well and incubated for 3 (FBS) or 4 days (hPL) and then in adipogenic differentiation medium (ThermoFisher Scientific, USA), and every third day a half change of media was performed. After 21 days, cells were fixed with formalin and stained with Oil Red O and hematoxylin (both Sigma Aldrich Chemie, Germany). Images were taken via an Eclipse TE300 microscope (Nikon, Japan). The intracellular Oil Red O was extracted by isopropanol (Carl Roth, Germany) by following the instructions from Ramírez‐Zacarías et al. [31], and the extinction was measured at a wavelength of 492 nm by an Anthos Zenyth 340 r (Anthos Mikrosysteme GmbH, Germany).

### 2.4. Growth Kinetics

About 1 × 10^4^ ADSCs were seeded per well in a 24‐well plate (TPP, Switzerland) and cultivated over 7 days with and without a mc. Every 24 h, cells were harvested with 0.25% trypsin/EDTA (Biochrom, Germany) and counted by Coulter Counter Z2 (Beckmann Coulter, Germany).

### 2.5. Cell Cycle Analysis

About 7 × 10^5^ ADSCs were seeded in T75 cm^2^ culture flasks and cultivated over 3, 5, and 7 days with and without a mc. After harvesting cells with 0.25% trypsin/EDTA and a wash step with DPBS (PAN‐Biotech, Germany), cells were stored in ethanol on ice. ADSCs were treated with RNase and propidium iodide (both Sigma Aldrich Chemie, Germany) and analyzed via flow cytometry.

### 2.6. Characterization of Spheres

#### 2.6.1. Sphere Formation

For the first attempt, ADSCs were seeded at high (2 × 10^4^, 3 × 10^4^, 4 × 10^4^, and 5 × 10^4^ cells per well), middle (5 × 10^3^, 1 × 10^4^, 1.5 × 10^4^ cells per well), and low (1 × 10^1^, 5 × 10^1^, and 1 × 10^3^ cells per well) densities in 10% FBS and hPL culture medium. For the second attempt, ADSCs were seeded at 1.5 × 10^4^ cells per well with different serum concentrations (0%, 2,5%, 5%, 10%, and 20%) of FBS or hPL. Every 24 h, cells were observed microscopically, and pictures of spheres were taken to determine their length and width.

#### 2.6.2. Von Kossa and Alcain Blue Staining

After seeding of 7.5 × 10^4^ cells per well in a 6‐well plate and 13 days incubation in hPL culture medium, ADSCs were fixed by 4% formaldehyde and stained according to the manufacturer’s instructions for von Kossa (Abcam, UK) and Alcain blue staining (Sigma Aldrich Chemie, Germany).

#### 2.6.3. SSEA‐3

Cells in passages 2 and 5 were cultivated over 7 days as a monolayer, and for spheres, 7.5 × 10^4^ cells were seeded per well and cultivated over 10 days. After harvest with 0.25% trypsin/EDTA and for spheres used Dispase (Stemcell Technologies, Canada), a wash step with stain buffer (BD Bioscience, Germany) and incubation with FITC antihuman/mouse SSEA‐3 and as‐isotype control FITC rat IgM (both Biolegend, USA) on ice, a further wash step and addition of 7‐AAD cells were measured by a flow cytometer Cytomics FC 500.

### 2.7. Statistical Analysis

All data were presented as the mean ± standard deviation (SD). The normality of the distribution of each parameter was assessed using the Anderson–Darling test. To identify differences between data sets, the two‐tailed Student’s *t*‐test was performed. Significance was assessed at *p* ≤ 0.05 ( ^∗^ *p* ≤ 0.05;  ^∗∗^ *p* ≤ 0.01; and  ^∗∗∗^ *p* ≤ 0.001). For comparative analysis of one dataset to a fixed value, the one‐sample *t*‐test was used. Here, a *p*‐value of ≤0.02 was considered a significant difference ( ^∗^ *p* ≤ 0.02;  ^∗∗^ *p* ≤ 0.01; and  ^∗∗∗^ *p* ≤ 0.002). For nonnormal distributed data to identify differences between data sets, the Mann–Whitney *U* test was performed. Significance was assessed at *p* ≤ 0.05 ( ^∗^ *p* ≤ 0.05).

## 3. Results

As described in our previous work, the isolated ADSCs meet the minimal criteria for the declaration of multipotent mesenchymal stromal cells [30], defined by the position statement of the ISCT in 2006 [2]. In addition, the experimental reproducibility was improved by a pooling procedure of ADSCs from human reduction mammoplasties of 10 different healthy female donors [32]. We have already demonstrated that the investigation results of pooled ADSCs (*n* = 10) are comparable to the average of the single analysis of each donor [32]. So, in the present study, pooled ADSCs were used for experiments.

### 3.1. Characteristics of ADSCs

ADSCs represent multipotent progenitor cells that have been isolated from adipose tissue and are characterized by certain morphology, surface antigen expression, a high rate of proliferation, and a diverse potential for differentiation [1]. Therefore, in the first approach, these attributes were analyzed in ADSCs that were cultured in hPL.

#### 3.1.1. Morphology of ADSCs Changed During hPL Cultivation, Immunophenotype

ADSCs are defined by their spindle‐shaped and fibroblast‐like morphology as well as their specific panel of expressed and lacking surface antigens (CD29^+^, CD90^+^, CD31^−^, CD34^−^, CD45^−^, and CD106^−^) [1, 3].

**Figure 1:**
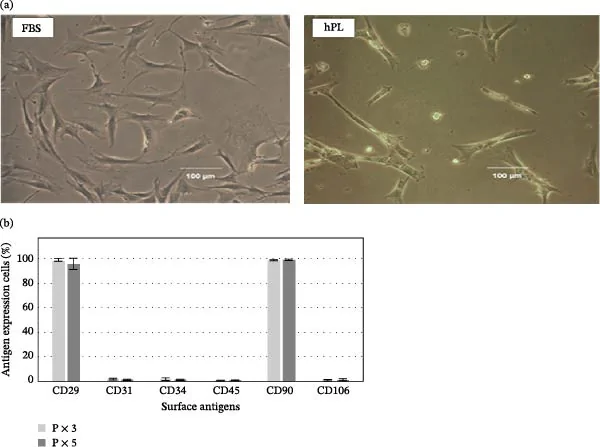
Morphology and surface antigen expression of ADSCs after 48 h of incubation. (a) Exemplary microscopic representation of ADSCs cultured in medium supplemented with fetal bovine serum (FBS) or human platelet lysate (hPL); magnification 200×, scale bar: 100 µm; (b) Surface antigen expression of ADSCs from passage three (Px3) and five (Px5) cultured in hPL. The expression values of isotype‐matched control immunoglobulin‐labeled cells were deducted from determined expression levels of each surface antigen, and unstained cells were also carried as controls. Data are presented as mean ± SD, *n* = 3.

The typical spindle‐shaped morphology appeared very homogeneous. In the hPL‐containing culture medium, the ADSCs also showed a spindle‐shaped appearance but with longer and narrower cell bodies than in the FBS culture (Figure 1a). A quantitative analysis from 50 ADSCs after 2 days of incubation shows a cell size of 194.04 ± 53.65 µm in FBS, 112 ± 39.27 µm in 10% hPL, and a size of 137.97 ± 61.64 µm in 2.5% hPL (see Supporting information Figure S1). The specific expression pattern of surface proteins did not change during the culture in hPL‐containing medium in comparison to FBS cultivation (Figure 1b), independently of passages (three or five).

#### 3.1.2. Improved Adipogenic Differentiation Capacity

**Figure 2:**
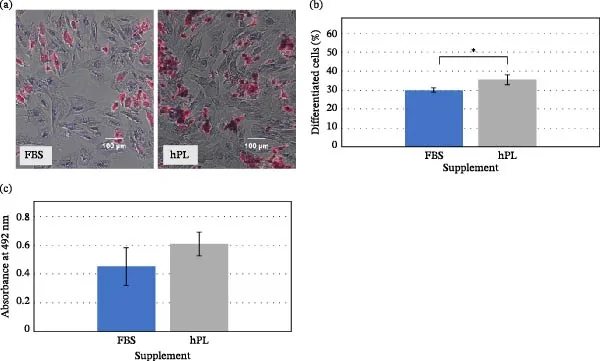
Adipogenic differentiation capacity of ADSCs in passage four cultured in DMEM/F12 supplemented with human platelet lysate (hPL) or fetal bovine serum (FBS). (a) Differentiated adipocytes were stained with Oil Red O (red; fat acids and triglycerides) and hematoxylin (blue; cell nuclei), magnification 100×, scale bar: 100 µm. (b) Ratio of Oil Red O‐stained ADSCs to unstained ADSCs in FBS‐ and hPL‐containing culture medium, counted microscopically, shown as mean ± SD, *n* = 3. (c) The intracellular Oil Red O was extracted with isopropanol, and the absorbance was determined at a wavelength of 492 nm, presented as mean ± SD, *n* = 3,  ^∗^ *p* ≤ 0.05 (two‐tailed *t*‐test).

The ADSCs cultured in FBS (ADSCs‐FBS) or hPL (ADSCs‐hPL) maintained their adipogenic differentiation capabilities after the application of appropriate induction media. To quantify and compare the differentiation rate of both approaches, adipocytes were stained by an Oil Red O solution (Figure 2a) and counted optically via microscopy (Figure 2b). Additionally, the amount of intracellularly absorbed dye was extracted and determined (Figure 2c).

About 120 h after incubation with adipogenic differentiation medium, ADSCs‐FBS started to form intracellular lipid droplets; in hPL‐ADSCs, this process began 24 h earlier. Furthermore, the overall ratio of differentiated cells was higher in hPL‐ADSCs (*p* ≤ 0.05, hPL: 35.5% vs. FBS: 30.1%). This is supported by the trend of higher amounts of extracted Oil Red O dye from differentiated ADSCs that were previously cultivated in hPL (Figure 2c; hPL: 0.61 vs. FBS: 0.45).

### 3.2. Growth Kinetics

#### 3.2.1. Enhanced Cell Growth of ADSCs‐hPL Containing Culture Medium

**Figure 3:**
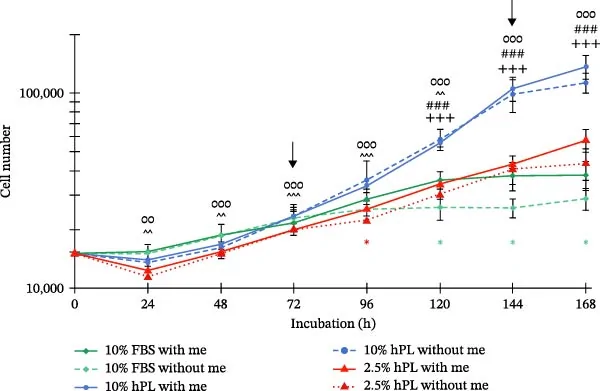
Growth kinetic of ADSCs in dependence of the culture medium supplements. ADSCs were cultured in DMEM/F12 supplemented with fetal bovine serum (FBS) or different concentrations of human platelet lysate (hPL) over 168 h with or without a medium exchange (me) after 72 and 144 h. The number of cells was determined every 24 h. Data are presented as mean ± SD from *n* = 5 (10% FBS with me), *n* = 4 (10% FBS without me; 2.5% hPL with me), *n* = 3 (10% hPL with me; 10% hPL without me; 2.5% hPL with me); significances were calculated for ADSCs in 2.5% hPL with me vs. ADSCs in 2.5% hPL without me (red):  ^∗^ *p* ≤ 0.05; ADSCs in FBS with me vs. ADSCs in FBS without me (green):  ^∗^ *p* ≤ 0.05; ADSCs in FBS with me vs. ADSCs in 10% hPL with me (#): ###*p* ≤ 0.001; ADSCs in FBS without me vs. ADSCs in 10% hPL without me (+): +++*p* ≤ 0.001; ADSCs in 2.5% hPL with me vs. in 10% hPL with me (^): ^^*p* ≤ 0.01, ^^^*p* ≤ 0.001; 2.5% hPL without me vs. in 10% hPL without me (°): °°*p* ≤ 0.01, °°°*p* ≤ 0.001 (two‐tailed *t*‐test).

The growth kinetics was investigated using daily cell counts of ADSCs‐hPL‐ or ‐FBS‐containing culture medium, whereby a medium exchange was performed every 3 days or not (Figure 3).

The growth of ADSCs‐FBS (10 % FBS) entered the lag phase within the first 24 h after seeding. The logarithmic phase began at 48 h and ended after 120 h with a population doubling time (PDT) of 52.37 h (PDTs, see Supporting information, Table T1). At 120 h, the proliferation stagnated and reached a plateau. When mcs were omitted, the logarithmic phase ended just 96 h after seeding, and PDT increased to 63.35 h. Thereafter, a stagnation of cell growth was visible at 96 h. In comparison to this, ADSCs‐hPL (10% hPL) were characterized by an enhanced cell growth rate (PDT FBS: 52.37 h vs. PDT hPL: 36.55 h), whereby a medium exchange was not necessary in order to achieve a continuous logarithmic growth over 7 days. Lower hPL concentrations resulted in a decrease in cell growth, which was reflected in an increased PDT from 36.55 to 65.48 h (with a mc) or 70.14 h (without a mc).

#### 3.2.2. Cell Cycle Distribution

**Figure 4:**
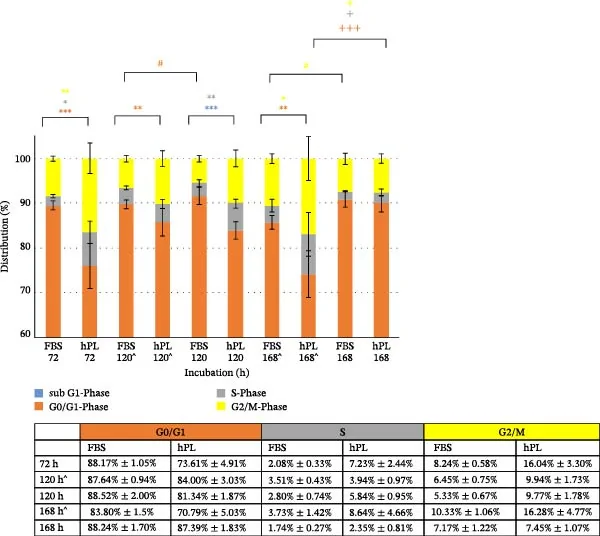
Cell cycle distribution of ADSCs cultured in FBS or hPL‐supplemented culture medium. ADSCs were cultured in DMEM/F12 supplemented with 10% fetal bovine serum (FBS) or 10% human platelet lysate (hPL). A medium exchange took place (^) or not after 72 and 144 h of cultivation. The distribution of cells in cell cycle phases (G0/G1, S, and G2/M) and cells with degraded cell DNA (subG1) was determined by flow cytometry using propidium iodide to measure the DNA content of each cell. Data are presented as mean ± SD from *n* = 3 (FBS), *n* = 4 (hPL 120 h, hPL 168 h without medium exchange), and *n* = 5 (hPL 72 h, hPL 168 h without medium exchange). Asterisks illustrate significant differences of ADSCs cultured in FBS or hPL:  ^∗^ *p* ≤ 0.05;  ^∗∗^ *p* ≤ 0.01; and  ^∗∗∗^ *p* ≤ 0.001; ADSCs in FBS with mc vs. ADSCs in FBS without mc (#): #*p* ≤ 0.05; ADSCs in hPL with mc vs. without mc (+): +*p* ≤ 0.05; +++*p* ≤ 0.001 (two‐tailed *t*‐test).

The observed differences in the growth kinetics of ADSCs‐hPL and ADSCs‐FBS were related to an altered distribution of the cells in the cell cycle phases (Figure 4). Example graphs of a flow cytometric measurement to determine the cell cycle phases are shown in Supporting Information, Figure S2.

In detail, at the time point of 72 h after seeding, the cultivation of ADSCs in hPL instead of FBS resulted in an increased rate of cells in the S phase (hPL: 7.23% vs. FBS: 2.08%, *p* ≤ 0.05) and G2/M phase (hPL: 16.04% vs. FBS: 8.24%, *p* ≤ 0.05), whereas the percentage of cells in the G0/G1 phase was decreased (hPL: 73.61% vs. FBS 88.17%, *p* ≤ 0.01). The renewal of the medium enhanced this trend (Figure 4). However, in order to maintain a stable ratio of cells in the S phase, a medium exchange was only necessary after 168 h in the hPL culture. Rather, this exchange after 72 h led to a short‐term inhibition of the cell cycle progression as the percentage of cells in the S phase decreased from 7.23% (72 h) to 3.94% (120 h with mc).

### 3.3. Characterization of Spheres

#### 3.3.1. Sphere Formation

**Figure 5:**
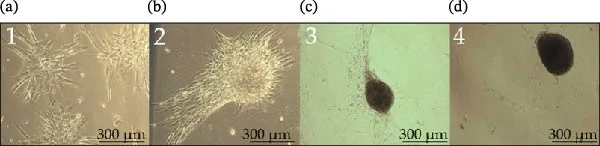
Stages of sphere formation of ADSCs: Microscopic representation of the ADSCs in Passage 5 cultivated in DMEM/F12 supplemented with hPL. (a) Stage 1—star‐shaped arrangement of the ADSCs at the bottom of the well; (b) Stage 2—heap‐like accumulation of ADSCs at the bottom of the well; (c) Stage 3—spherical but still‐adherent sphere at the bottom of the well; and (d) Stage 4—free‐floating sphere in the culture medium; magnification 100×, scale bars: 300 µm.

During cultivation with FBS and hPL, differences in the growth patterns of ADSCs were observed. In FBS‐containing culture medium the cells grew flat next to each other in 2D (shown in Supporting Information, Figure S3). When cultured in hPL‐supplemented medium, ADSCs formed spontaneous three‐dimensional (3D) structures (spheres, Figure 5).

After 48 h of cultivation, adherent cells migrated groupwise to each other and were arranged in a star‐shaped conformation (Figure 5a). Afterwards, these 2D complexes started to overlap and organized in a 3D heap‐like accumulation (Figure 5b) till a spheroid had been built (Figure 5c), which in a last step separated from the culture dish bottom and floated within the culture medium (Figure 5d). Because of the morphological similarity, a spontaneous differentiation into osteo‐ or chondrocytes was excluded via von Kossa stain (osteocytes) and Alcian Blue staining (chondrocytes); (Supporting Information, Figure S4).

**Figure 6:**
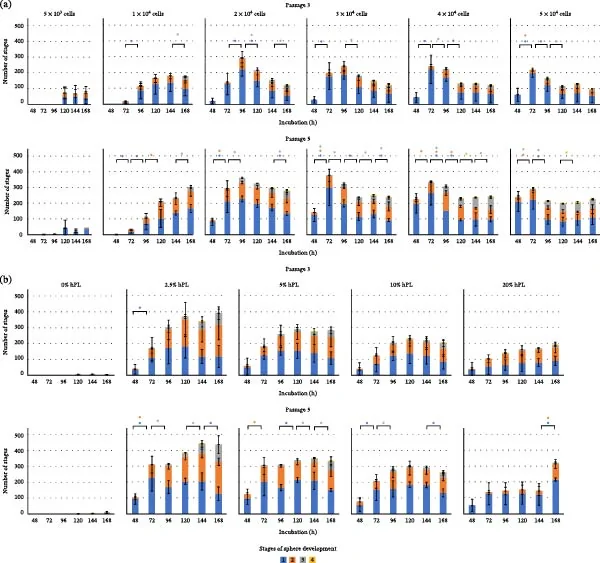
Time‐dependent development of sphere formation of ADSCs of passage three and five in (a) depending on seeding density, cultivated in 10% hPL‐containing medium, and (b) cultivated in different hPL concentrations (seeding density 1.5 × 10^4^ ADSCs/well). Numbers shown as mean ± SD (*n* = 3); blue = stage 1: star‐shaped arrangement of the ADSCs at the bottom of the well; orange = stage 2: heap‐like accumulation of ADSCs at the bottom of the well; gray = stage 3: spherical but still‐adherent sphere at the bottom of the well; yellow = stage 4: free‐floating sphere in the culture medium. Significances were calculated for each specific sphere stage in comparison to various time points and are shown as  ^∗^ *p* ≤ 0.05 (Mann–Whitney *U*‐test).

To further characterize spontaneous sphere formation in hPL, ADSCs were analyzed over 7 days under different culture conditions, such as passage number, seeding densities (Figure 6a), and hPL concentrations (Figure 6b). The number of precursors and spheres formed was determined on each day of incubation.

At seeding densities of 2 × 10^4^ and higher, ADSC sphere formation began after just 48 h of incubation (Figure 6a). In contrast, at lower seeding densities (5 × 10^3^ and 1 × 10^4^), sphere formation was delayed. Stage 1 spheres were most frequently observed, followed by stages 2 and 3. Stage 4 formation of ADSCs was the least frequently counted during analysis. Without the addition of hPL to the medium, hardly any sphere formation was detectable (Figure 6b). Sphere formation was most pronounced in ADSCs cultured in a 2.5% hPL. With increasing hPL concentrations in the medium (5%, 10%, and 20%), sphere formation steadily decreased. When comparing the two passages of the ADSCs, it was noticeable that the sphere formation in passages 3 and 5 showed similar distribution patterns. However, more spheres were detectable overall in the higher passage (Figure 6a,b).

#### 3.3.2. SSEA‐3 Subpopulation in ADSCs

**Figure 7:**
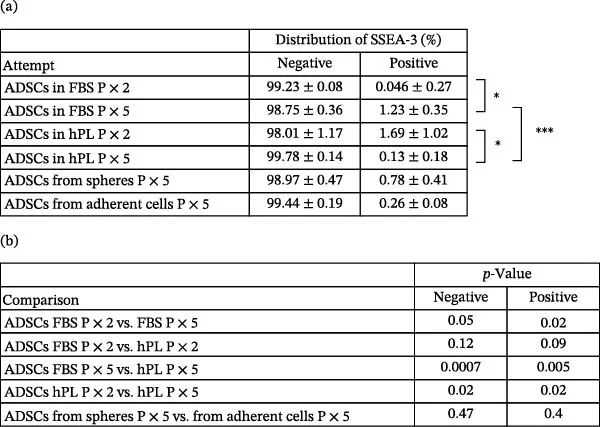
SSEA‐3 expression [%] in the total population of ADSCs: (a) percentage distribution of SSEA‐3 expressing (positive) and non‐SSEA‐3 expressing (negative) ADSCs from passage two (Px2) and five (Px5) in 10% FBS and 10% hPL‐containing culture medium, presented as mean ± SD from *n* = 4 (FBS Px2; hPL Px2), *n* = 5 (FBS Px5; hPL Px5), *n* = 3 (ADSCs from spheres Px5; ADSCs not from spheres, Px5); Significances are shown as  ^∗^ *p* ≤ 0.05,  ^∗∗∗^ *p* ≤ 0.001 (two‐tailed *t*‐test); (b) comparison of attempts for significance calculation indicating the *p*‐values.

SSEA‐3 is expressed by a subpopulation of ADSCs, the so‐called MUSE cells. These cells are thought to be responsible for initiating sphere formation. Therefore, we investigated whether this subpopulation could be detected in ADSCs from passages two and five (Figure 7). Example graphs of a flow cytometric measurement to determine the SSEA‐3 expression are shown in Supporting Information, Figure S5. Furthermore, it was examined whether the proportion of MUSE cells changed depending on the medium supplement (FBS and hPL) in the spheres and in the still‐adherent ADSCs, which were not part of the sphere.

ADSCs from human breast expressed SSEA‐3 in both FBS‐ and hPL‐supplemented medium. The proportion of SSEA‐3‐positive cells was 0.46% (FBS) and 1.69% (hPL) at passage 2 but 1.23% (FBS) and 0.13% (hPL) at passage 5. Spheres cultured in hPL at passage 5 contained 0.78% SSEA‐3‐positive cells. However, the adherent cells also contained 0.26% SSEA‐3‐positive ADSCs.

## 4. Discussion

In this study, the effects of hPL compared to FBS as a culture medium supplement for ADSCs isolated from the female breast tissue were analyzed. Existing studies have only addressed the use of hPL for ADSCs obtained from the abdominal adipose tissue. As outlined in the Introduction section, ADSCs from breast tissue exhibit certain distinct characteristics compared to those from abdominal fat, due in part to their origin and the differing microenvironment.

In FBS‐ and hPL‐containing culture media, the breast ADSCs used in our study exhibited different morphologies. The cells appeared as distinctly narrow, spindle‐shaped cell bodies with longer cell processes in hPL‐containing culture medium compared to those in FBS‐containing culture medium. Similar morphological changes were also described for ADSCs from abdominal fat tissue in hPL‐supplemented medium [33, 34]. The specific spectrum of expressed and absent surface antigens (CD29^+^, CD90^+^, CD31^−^, CD34^−^, CD45^−^, and CD106^−^) did not change during culture in hPL‐containing medium compared to FBS culture (described in [30]), regardless of passages (three or five). In the literature, there is also no change in the immunophenotype of ADSCs, but from human surplus abdominal adipose tissue, after hPL culture compared to FBS culture, could be observed [35, 36].

This study also showed the same immunophenotype (CD29^+^, CD90^+^, CD31^−^, CD34^−^, CD45^−^, and CD106^−^) of ADSCS from the breast as previously described [27, 37]. According to the ISCT guidelines, the following CD expression pattern is described for ADSCs: CD73, CD90, and CD105 positive (over 95%); CD45, CD34, CD14, or CD11b, CD79a, or CD19, and HLA‐DR negative (under 2%) [2]. However, some research groups describe a variable CD106 expression in ADSCs—for instance, those derived from abdominal adipose tissue—ranging from weakly positive to negative; this constitutes another distinguishing feature compared to bone marrow‐derived MSCs, which are CD106‐positive [38–40].

In addition to unlimited growth, stem cells are characterized by their ability to differentiate. The breast‐derived ADSCs used in this study retained their ability for adipogenic differentiation in hPL‐containing culture medium. They even showed a greater differentiation capacity than that of ADSCs‐FBS. Not only did the formation of intracellular lipid droplets begin 24 h earlier in the hPL‐containing culture medium than in ADSCs‐FBS, but the total proportion of differentiated cells in hPL‐ADSCs was also significantly higher. A more pronounced degree of adipogenic differentiation under hPL compared to FBS has also been described for abdominal fat ADSCs [25, 41].

ADSCs cultured with hPL (10%) exhibited an increased cell growth rate. The PDT was 36.55 h, compared to 52.37 h for ADSCs cultured with FBS (10%). The mcs were not necessary to achieve continuous logarithmic growth over 7 days. A lower hPL concentration (2.5% hPL) led to a reduction in the cell growth rate, which was reflected in a prolongation of PDT from 36.55 h (10% hPL) to 65.48 h (with mcs) and 70.14 h (without mcs). Similar results of shortened PDT for ADSCs in a hPL‐containing culture have also been reported for ADSCs from abdominal fat. For example, Cheng et al. (2020) [36] described a decreased PDT for ADSCs from subcutaneous fat tissue of 43.0 h (1% hPL), 32.1 h (2% hPL), and 28.6 h (5% hPL) compared to 58.0 h (10% FBS). Kølle et al. [42] described a PDT of 29.6 h from abdominal ADSCs. This was also observed by Ballesteros et al. [25]. They also demonstrated decreasing PDT with increasing hPL concentrations in ADSCs isolated from lipoaspirates (5% hPL: 100.9 h; 10% hPL: 50.5 h; and 20% hPL: 35.6 h). However, the PDT of ADSCs derived from abdominal adipose tissue described in the literature is clearly lower than that of the ADSCs derived from breast tissue used in the present study.

Due to these growth kinetics of ADSCs in hPL‐containing cultures, significantly higher cell counts were achieved than in FBS‐containing cultures despite the same seeding densities (10% hPL with mc: 13.6 × 10^4^ cells vs. 10% FBS with mc: 3.7 × 10^4^ cells; 10% hPL without mc: 11.3 × 10^4^ cells vs. 10% FBS without mc: 2.8 × 10^4^). Even lowering the hPL concentration to 2.5% hPL still resulted in significantly higher cell counts than when using 10% FBS. This demonstrates an overall positive effect of hPL on ADSC proliferation. This is crucial for therapeutic applications. If a sufficient number of cells can be generated in a short interval, delays in the treatment process can be avoided. For example, in stem cell therapy, MSCs are extracted from the bone marrow, peripheral blood, or umbilical cord, processed in vitro, and administered to the patient [43]. To achieve a promising therapeutic outcome, at least 2 × 10^6^ stem cells per kilogram of patient body weight are required [44]. Furthermore, it can be argued that cultivation with hPL requires less labor (medium renewal can be skipped for 168 h) and requires less material, thus saving costs. If no medium was renewed in the culture with FBS on the third day, the logarithmic growth of the ADSCs ceased, and the plateau phase was reached. This means that the cells are no longer capable of mitosis and remain in the G1 phase. This was also reflected in the cell cycle analysis: after 72 h of cultivation, 88.17% of the cells cultured with FBS were in the G1 phase, 2.08% in the S phase, and 8.24% in the G2/M phase. In contrast, in the cells of the hPL culture, approximately three times as many cells were in the S phase and twice as many cells were in the G2/M phase. Therefore, a medium exchange must be performed after 3 days for ADSCs in FBS to increase the concentration of growth factors and remove toxic cell degradation products. In cultivation with hPL, no mc was necessary after 3 days as significantly more cells were in the S phase. This may be due to the increased content of growth factors in hPL and the resulting higher growth stimulus. Kakudo et al. [24] also described a strong proliferative impulse in ADSCs. They conducted cell cycle analyses with 5% hPL and 10% FBS in the culture medium. The authors found an increased number of cells in the S and G2/M phases in the hPL‐containing culture.

During cultivation in hPL, our study showed sphere formation of ADSCs after ~96 h. This occurred spontaneously and without any additional aid. However, in the FBS‐containing culture, no sphere formation was observed, even after varying the culture density and serum concentration. Sphere formation has been described in the literature using various methods, such as the spinner flask method, the hanging drop method, and low‐attachment plates. Depending on the type, application, and cell number, spheres of varying sizes were generated. In our study, ADSCs formed spheres in hPL‐containing medium without the need for additional sphere generation methods or additional growth factor administration. The cells grew as an adherent monolayer and then clustered in a star shape and formed clusters, from which spheres formed and floated freely in the medium. However, in the FBS‐containing culture, no sphere formation was observed even after varying the seeding density and serum concentration. A spontaneous differentiation into osteo‐ or chondrocytes could be excluded. We could observe that the size of the spheres could be influenced by the hPL concentration and seeding density. Contrary to the assumption that higher hPL concentrations (10% and 20% hPL) lead to an increased number of spheres, this study showed an increased number of spheres at low hPL concentrations (2.5% and 5% hPL). This inverse dose–response relationship regarding sphere formation at higher hPL concentrations (optimum at 2.5%) contrasts with the dose‐dependent proliferative advantage of higher hPL concentrations (10%) demonstrated by growth kinetics and cell cycle data. Based on current knowledge, there is no plausible explanation for this. Some studies add growth factors such as PDGF‐BB, VEGF, and HGF to the culture medium to induce sphere formation [45]. Compared to FBS, hPL contains higher overall concentrations of growth factors. It can, therefore, not only increase ADSC proliferation but also trigger the initiation of sphere formation. Schallmoser et al. [44], Shanskii et al. [46], and Duarte Rojas et al. [47] conducted analyses of various components of FBS and hPL. They found that growth factors, such as basic fibroblast growth factor (bFGF), epidermal growth factor (EGF), platelet‐derived growth factor (PDGF and its isoforms AA, AB, and BB), and tissue growth factor ß (TGF‐ß), were present in significantly higher concentrations in hPL than in FBS. According to the manufacturer’s information, the hPL used in this study also contained concentrations of human EGF, bFGF, and PDGF‐AB that were comparable to the data from Schallmoser et al. [44] and Shanskii et al. [46], respectively. This indicates that further investigations—for example, at the molecular level—regarding sphere formation are required to rule out the possibility that the high proliferative effect of hPL is driven by early lineage differentiation of ADSCs [22].

Spherically derived MSCs are described as more promising for therapeutic applications due to their higher differentiation capacity and stronger anti‐inflammatory, angiogenic, and promitotic effects [48, 49]. Therefore, the cultivation of MSCs as spheres is receiving considerable interest, including for the treatment of ischemic diseases of the brain and cardiovascular system [50]. Similarly, a better survival rate was observed after injection of MSCs cultured as spheres as they have already experienced hypoxic conditions during in vitro cultivation [51]. This highlights the importance of identifying the precise mechanisms of sphere formation so that they can be used for therapeutic purposes [50]. Further investigations should also be carried out regarding immunophenotype, gene expression levels for pluripotent markers (NANOG, OCT‐4, and SOX‐2) or the amount of cytokine release, or the potential of differentiation to compare the spontaneously developed spheres to spheres produced with methods or low‐attachment plates.

SSEA‐3‐expressing cells—multilineage‐differentiating stress‐enduring cells (MUSE cells)—are discussed as the initiator of sphere formation [10]. It has been shown that ADSCs from mammary adipose tissue also express SSEA‐3 at less than 3%, just like MSCs from bone marrow [13]. Likely wise, in commercially available ADSCs, the proportion of Muse cells is 3.8% ± 0.9% [52]; in contrast, the proportion of Muse cells isolated freshly from abdominal adipose tissue ranges from 1.91% ± 0.42% [53] to 8.8% ± 1.3% from freshly isolated subcutaneous adipose tissue [52]. The presence of MUSE cells in the spheres was also demonstrated in our study. But it cannot be clearly stated whether they represent the starting function of sphere formation since another portion of the MUSE cells was found in the adherently growing ADSCs at the well bottom. MUSE cells can differentiate into tissue types of all three germ layers without degenerating into malignant tumors. This differentiation capacity can be used for therapeutic approaches and cell‐based therapies [54]. For example, differentiation into hepatocytes has been demonstrated, which can be used to treat liver cirrhosis. Similarly, MUSE cells show promising properties for the cell‐based therapy of diabetic wounds in mouse models [53]. Over and above that, MUSE cells combined, for example, with hyaluronic acid‐based dissolvable microneedles containing salvianolic acid, offer a potential treatment for hypertrophic scars [55]. Additionally, administered nanoparticles containing green tea polyphenols (PPs) and epigallocatechin‐3‐gallate (EG) can increase the apoptosis rate of breast cancer cells (MCF7, MDA‐MB‐231) [56].

## 5. Conclusions

Overall, based on the data presented, we were able to demonstrate that hPL as a medium supplement represents a promising alternative to FBS in the cell culture of breast ADSCs. Thus, its use is conceivable not only in in vitro expansion for clinical therapies but also in the field of tissue engineering. Further research on this topic is needed. We were also able to demonstrate that using hPL makes it possible to generate ADSC spheres without the need for special methods like the hanging drop method or coating wells with agarose or material like a low‐attachment plate that externally forces sphere formation. However, further investigations into sphere formation are needed.

## Funding

This research was funded by a grant from the B. Braun Foundation (Grant BBST‐2018‐D‐18‐00030), Melsungen, Germany. Open Access funding enabled and organized by Projekt DEAL.

## Conflicts of Interest

The authors declare no conflicts of interest.

## Supporting Information

Additional supporting information can be found online in the Supporting Information section.

## Supporting information


**Supporting Information** The Supporting Information file with Table T1 (population doubling times of ADSCs in FBS‐ and hPL‐containing culture media), Figures S1 (example graph of a flow cytometric measurement to determine the cell cycle phases of ADSCs), S2 (exclusion of spontaneous differentiation of ADSCs into osteo‐ or chondrocytes), and S3 (example graph of a flow cytometric measurement to determine the SSEA‐3 expression from ADSCs in FBS‐ or hPL‐containing culture medium) is provided in the Supporting Information.

## Data Availability

The data underlying this article will be shared upon reasonable request to the corresponding author.
