# Supplementary material for: The Use of Human Platelet Lysate (hPL) Enables 3D Cultivation of ADSCs Derived From Human Breast Tissue
Source: Stem Cells Int. 2026 Sep 22;2026:3165313. doi: 10.1155/sci/3165313 (PMC13598434; doi:10.1155/sci/3165313)
Supplement: Supplementary file 1 [file SCI-2026-3165313-s001.docx]

**Supplements**

**Table T1**

**Table T1:** **Population doubling times (PDT) of ADSCs in FBS- and hPL-containing culture media** with and without medium change (mc) after 72 h and 144 h of incubation, determined by exponential regression analysis in the range of log growth, presented as mean ± standard deviation from n=5 (10% FBS with mc), n=4 (10% FBS without mc; 2.5% hPL with mc), n=3 (10% hPL with mc; 10% hPL without mc; 2.5% without mc); FBS: fetal bovine serum; hPL: human platelet lysate; mc: medium change.

| **Attempt** | **range of log growth** | **PDT** |
| --- | --- | --- |
| 10 % FBS with mc | 48 h - 120 h | 52,37 ± 5,92 h |
| 10 % FBS without mc | 24 h - 96 h | 63,35 ± 8,57 h |
| 10 % hPL with mc | 48 h - 144 h | 36,55 ± 3,51 h |
| 10 % hPL ohne mc | 48 h - 144 h | 37,59 ± 5,12 h |
| 2,5 % hPL without mc | 48 h - 144 h | 65,48 ± 6,51 h |
| 2,5 % hPL ohne mc | 48 h - 144 h | 70,14 ± 14,17 h |

**Figure S1**

**Figure S1: Mean size of ADSCs cultivated in media containing FBS or hPL** after an incubation time of 48 hours. Results are presented as mean ± standard deviation from n=50.

**Figure S2**

**Figure S2:** Example graph of a flow cytometric measurement to determine the cell cycle phases (sub G1, G0/G1, S and G2/M) of (a) ADSCs in FBS-containing culture medium in passage four after 168 h incubation without a medium change; (b) ADSCs in hPL-containing culture medium in passage five after 72 h incubation.

**Figure S3**

**Figure S3: ADSCs in FBS-containing culture media** at various time points during incubation at 100× magnification; scale bar: 100 µm.

**Figure S4**

**Figure S4: Exclusion of spontaneous differentiation of ADSCs into osteo- or chondrocytes:** Developmental stages in the sphere formation of ADSCs after **(a)** Alcian Blue staining and **(b)** after von Kossa stain and subsequent nuclear fast red staining; each at 100x magnification; scale bar: 100 µm; stages: (1) starshaped arrangement; (2) cluster-like aggregation of cells; (3) spherical, adherent sphere. Alcian Blue stains sulfated glycosaminoglyclanes, which are located on the surface of chondrocytes, cyan (a). In samples no blue staining could be observed. A von Kossa stain dyes extracellular calcium deposits black; these deposits arise during prior differentiation into osteocytes. No accumulations of gray to black stained extracellular calcium deposits were observed in samples (b).

**Figure S5**

**Figure S5**: Example graph of a flow cytometric measurement to determine the SSEA-3 expression from (a) ADSCs in FBS-containing culture medium in passage five; (b) ADSCs in hPL-containing culture medium in passage two.
